# Whole-genome sequencing reveals high-risk ESBL and carbapenemase-producing *Enterobacterales* on maternity ward surfaces in Cameroon

**DOI:** 10.1038/s41598-026-54772-x

**Published:** 2026-06-05

**Authors:** Gabriel Cedric Bessala, Germanie Delaisie Abomo, Henri Rodrigue Njengoue Ngamaleu, Felix Essiben, Nicole E. Wheeler, Michelle M. C. Buckner, Jan-Ulrich Kreft, Blaise Pascal Bougnom

**Affiliations:** 1 Department of Microbiology, Faculty of Science, University of Yaounde 1, Yaounde, Cameroon; 2 Centre for Research in Infectious Diseases (CRID), Yaounde, Cameroon; 3 Faculty of Education, University of Yaounde 1, Yaounde, Cameroon; 4 Department of Obstetrics and Gynecology, Central Hospital of Yaounde, Yaounde, Cameroon; 5 Institute of Microbiology and Infection, University of Birmingham, Birmingham, UK; 6 Department of Microbes, Infection, and Microbiomes, College of Medicine and Health, University of Birmingham, Birmingham, UK; 7 School of Biosciences, University of Birmingham, Birmingham, UK

**Keywords:** Antibiotic resistance, Maternity wards, ESBL, Carbapenemases, *Escherichia coli*, *Klebsiella* spp., Microbiology, Molecular biology

## Abstract

The hospital environment is increasingly recognized as a critical reservoir for antimicrobial-resistant (AMR) bacteria. In sub-Saharan Africa, maternity wards represent high-risk settings where environmental contamination poses a direct threat to vulnerable mothers and neonates. Despite this, there is a significant lack of data integrating phenotypic resistance with whole-genome sequencing (WGS) to understand antimicrobial resistance (AMR) in these settings. This study characterized the AMR patterns and genomic features of ESBL-producing *Escherichia coli* and *Klebsiella* spp. isolated from maternity ward surfaces in Yaounde, Cameroon. A cross-sectional environmental study was conducted across four maternity wards. Isolates were identified via standard microbiological methods, and antimicrobial susceptibility testing against 13 antibiotics was performed following European Committee on Antimicrobial Susceptibility Testing (EUCAST) 2024 guidelines. Short-read WGS was utilized to identify sequence types (STs), plasmid incompatibility groups, antibiotic resistance genes (ARGs), and virulence factors. Plasmid-ARG association networks were constructed to visualize resistance dynamics. Nineteen ESBL-producing *Enterobacterales* were identified, comprising 15 *E. coli* and four *Klebsiella* spp. isolates. High levels of multidrug resistance were observed against ciprofloxacin, penicillins, and third-generation cephalosporins. While the isolates remained sensitive to colistin and imipenem, alarming resistance to meropenem was detected. Genomic analysis revealed the presence of globally disseminated high-risk lineages, including *E. coli* ST131, ST1193, and ST410, alongside *K. pneumoniae* ST1324 *and K. quasipneumoniae* ST489. Critical resistance determinants, including ESBLs, AmpC enzymes, and carbapenemases (*bla*_NDM_, *bla*_OXA-48-like_), were associated with epidemic plasmids such as IncF, IncA/C2, and IncL/M. Additionally, the isolates harboured virulence factors characteristic of extraintestinal pathogenic *Enterobacterales*. The widespread presence of high-risk carbapenemase-producing clones on maternity ward surfaces identifies the hospital environment as a significant AMR reservoir in Yaounde. These findings highlight the urgent need for reinforced infection prevention and control (IPC) measures, robust antimicrobial stewardship, and the integration of genomic surveillance to safeguard highly susceptible maternal and neonatal populations from life-threatening infections.

**Supplementary Information:**

The online version contains supplementary material available at 10.1038/s41598-026-54772-x.

## Introduction

Neonatal and maternal mortality are major health challenges facing Cameroon and other sub-Saharan African countries. Recent data indicate that the neonatal mortality rate in Cameroon is approximately 2,800 deaths per 100,000 live births^1^. While global maternal mortality has declined, the ratio remains high in Cameroon, with approximately 529 deaths per 100,000 live births^2^.

Neonatal infections such as sepsis, pneumonia, and healthcare-associated infections (HAIs) have been identified as the leading causes of neonatal death in low- and middle-income countries (LMICs)^3^. Consistent reports from sub-Saharan Africa show that maternal and neonatal sepsis are among the top causes of death, emphasizing the need to strengthen infection prevention in maternity and neonatal units^3,4^. Such epidemiological data highlight the importance of examining infection risks in maternity wards, where highly susceptible mothers and newborns are exposed to hospital environments during their most delicate times of care.

HAIs significantly cause sickness and death and increase healthcare costs worldwide, but the burden is disproportionately greater in LMICs where there is limited infection prevention and control (IPC) infrastructure, overcrowding, and insufficient surveillance^5,6^. Pathogens of concern include extended-spectrum beta-lactamase (ESBL)-producing *Escherichia coli* and *Klebsiella pneumoniae*, which are considered critical priority pathogens by the World Health Organization because of their multidrug resistance and role in severe invasive infections^7^. In Africa, these pathogens are frequently the cause of maternal and neonatal sepsis, as antimicrobial resistance (AMR) levels rise rapidly^8^. *K. pneumoniae* belongs to the ESKAPE pathogen group (*Enterococcus faecium*, *Staphylococcus aureus*, *K. pneumoniae*, *Acinetobacter baumannii*, *Pseudomonas aeruginosa*, and *Enterobacter* species). This group collectively accounts for a substantial proportion of HAIs worldwide and is defined by high levels of AMR, an increasingly limited therapeutic pipeline, and the capacity to escape the effects of currently available antibiotics^9^.

The hospital setting has been increasingly studied as a major reservoir and transmission route for AMR. Contaminated surfaces, medical devices, and healthcare workers’ hands play a role in the survival and indirect spread of multidrug-resistant bacteria^10^. Environmental contamination has been linked to the spread of ESBL-producing *Enterobacterales* in high-risk units such as intensive care and neonatal wards^11^. However, evidence from African hospitals remains limited and disjointed, especially in maternity settings.

Maternity wards represent some of the least researched areas in LMICs despite their contribution to HAIs due to high patient turnover and numerous invasive procedures^12^. Studies in sub-Saharan Africa have revealed ESBL-producing *E. coli* and *Klebsiella* spp. as hospital clinical isolates, including those from neonatal and obstetric units^13,14^. Despite this, very few comprehensive studies have integrated environmental sampling, phenotypic resistance profiling, and genomic characterization in these settings. A recent systematic review of carbapenem-resistant *Enterobacteriaceae* at the human-animal-environment interface in Africa found no eligible studies from Central Africa, highlighting a major regional evidence gap^15^. Whole-genome sequencing (WGS) allows the mapping of resistance genes and transmission mechanisms at a fine scale, but its usage remains largely unavailable in LMIC maternity wards^16^.

Thus, this investigation determined the extent of environmental contamination in maternity wards in Yaounde, Cameroon, by ESBL-producing *E. coli* and *Klebsiella* spp. By utilizing extensive environmental sampling and WGS, this work helps narrow the evidence gap concerning the environmental sources of AMR to reinforce data-driven IPC and antimicrobial stewardship (AMS) interventions.

## Materials and methods

### Study design and setting

The study was carried out in Yaoundé, the political capital of Cameroon. The city has more than four million inhabitants. Maternity wards in the following hospitals were investigated: Yaounde Central Hospital (HCY) in the Yaounde I district, Cite Verte district hospital (HDC) in the Yaounde V district, Nkolndongo district hospital (HDN) in the Yaounde III district, and Odza district hospital (HDO) in the Yaounde VI district. These healthcare facilities were chosen because they cover different types of wards found across diverse administrative districts of the city. This study was designed exclusively as an environmental surveillance investigation; no clinical isolates were included. Accordingly, all isolates analyzed originated from environmental surface samples.

### Sample collection

Environmental samples from the surfaces and air of the four maternity wards were collected monthly from January to November 2024. Monthly repeated sampling over 11 months captures both temporal and seasonal variation (wet and dry seasons in Cameroon), detecting the continuous circulation of high-risk STs across multiple sampling points. Sampling targeted nine functional areas that are commonly involved in patient care and staff activities, including (1) delivery rooms, (2) postpartum wards, (3) neonatal care units, (4) consultation rooms, (5) operating theaters, (6) corridors, (7) waiting areas, (8) sanitary facilities, and (9) waste zones. These areas were selected on the basis of their high frequency of human contact, potential microbial contamination, and association with IPC practices. To ensure the representativeness of the results, sampling was executed during routine clinical activity. All sampling material-handling techniques were aseptic, and in each session, field controls were taken to keep track of possible external contamination related to handling, transport, or background environmental sources.

Surface sampling was performed via sterile swabs premoistened with buffered peptone water. Swabbing was carried out on high-touch and high-risk surfaces such as bed rails, delivery tables, incubators, medical equipment, door handles, sinks, worktops, and waste container lids. Each surface was swabbed over an approximate area of 10 cm × 10 cm via a standardized multidirectional technique to maximize the recovery of microorganisms. After surface sampling, the swabs were immediately placed in sterile transport tubes.

Air sampling was conducted in delivery rooms, postpartum wards, and neonatal care units to assess airborne microbial contamination. Passive air sampling followed standard settling-plate procedures in which MacConkey agar plates supplemented with cefotaxime (2 mg/L) were exposed for 1 h at a height of 1 m above the floor and at least 1 m away from walls or obstacles^17^. Following exposure, the plates were sealed and labelled. Both swabs and plates were transported within four hours of collection in cool boxes (4–8°C) to the laboratory for processing.

### Isolation and identification of ESBL-producing *E. coli* and *Klebsiella* spp.

All the environmental surface and air samples were immediately processed upon arrival at the laboratory. The surface swabs were streaked onto MacConkey agar supplemented with 2 mg/L cefotaxime as a selective medium for the isolation of cefotaxime-resistant Gram-negative enteric bacteria. The plates harboring the air samples and streaked swabs were incubated at 37°C for 24 h, after which colonies showing the typical morphology of *E. coli* or *Klebsiella* spp. and fermenting lactose were selected for further identification. When more than one colony morphotype was present on a plate, representative colonies were purified to recover different resistant strains. Presumptive isolates were streaked onto CHROMagar™ ESBL medium (CHROMagar, Paris, France) to confirm resistance and provide preliminary species identification on the basis of colony color from hydrolysis of chromogenic substrates. All colonies with a coloration consistent with those specified in the manufacturer’s instruction leaflet to be indicative of ESBL-producing *E. coli* or *Klebsiella* spp. were subcultured until purity and identified through biochemical methods via the API 20E system (bioMérieux, Marcy-l’Etoile, France). The results were interpreted via the API database. All the isolates confirmed as ESBL-producing *E. coli* or *Klebsiella* spp. were stored in 20% glycerol stocks at -80°C for future antimicrobial susceptibility testing and WGS.

### Antimicrobial susceptibility testing (AST)

The antimicrobial susceptibility of all ESBL-producing *E. coli* and *Klebsiella* spp. was determined via the Kirby-Bauer disk diffusion method on Mueller–Hinton agar (MHA) in accordance with EUCAST 2024 recommendations^18^. Antibiotic discs were obtained from HiMedia Laboratories Pvt. Ltd. (Mumbai, India), and quality control was performed using *E. coli* ATCC 25,922 and *K. pneumoniae* ATCC 700,603. The turbidity of the bacterial suspensions was adjusted to the turbidity of a 0.5 McFarland standard; sterile swabs were used to inoculate MHA plates uniformly. The following antimicrobials were evaluated (per mL): amoxicillin (10 µg), amoxicillin-clavulanic acid (20/10 µg), cefoxitin (30 µg), ceftriaxone (30 µg), ceftazidime (30 µg), cefotaxime (30 µg), aztreonam (30 µg), meropenem (10 µg), imipenem (10 µg), gentamicin (10 µg), tobramycin (10 µg), ciprofloxacin (5 µg), and colistin (10 µg). This antibiotic panel was selected to represent the major antimicrobial classes relevant to ESBL-producing Enterobacterales, in accordance with EUCAST recommendations and international AMR surveillance priorities.

The antibiogram results were interpreted as resistant (R), susceptible (S), or susceptible at increased exposure (I) according to the EUCAST 2024 breakpoints.

### DNA extraction, library preparation, and whole-genome sequencing

DNA extraction, library preparation and WGS of all the ESBL-producing *E. coli* and *Klebsiella* spp. isolates were conducted at MicrobesNG, Birmingham, UK, on an Illumina HiSeq 2500 platform to generate 250 bp paired-end reads (min. 30 × coverage per genome)^19^.

### Bioinformatics and genomic analysis

Raw sequence reads were processed through the standardized bioinformatics pipeline of MicrobesNG, comprising an initial quality check of FASTQ files via FastQC v0.11.9 and removing low-quality bases together with adapter sequences via Trimmomatic v0.39^20^. The cleaned reads were de novo assembled via the St. Petersburg genome Assembler (SPAdes v3.15.5) with default parameters^21^. The assembly quality (N50, total length, and number of contigs) was checked via the Quality Assessment Tool (QUAST v5.2.0)^22^. Contigs shorter than 500 bp or with coverage less than 10 × were removed from further analysis. Species confirmation was achieved via Mash and Kraken2, whereas multilocus sequence typing (MLST) was performed via the mlst tool^23^ with the PubMLST database (https://pubmlst.org/). Plasmid replicons were detected in silico via PlasmidFinder, with thresholds set to ≥ 95% identity and ≥ 60% coverage^24^. Antibiotic resistance- and virulence-associated genes were detected via ABRicate against the database ResFinder v4.7.2, Comprehensive Antibiotic Resistance Database (CARD v3.3.0), ARG-ANNOT v6, and Virulence Factor Database (VFDB v6.0) with settings of ≥ 90% identity and ≥ 90% coverage^25^. For phylogenomic analysis, the genomes were initially annotated with Prokka v1.14.6^26^. Then, the pangenome analysis software Roary v3.13.0^27^ was used, with a ≥ 95% identity threshold for BLASTp, to generate the core-genome alignment for phylogeny. Maximum likelihood trees were generated with IQ-TREE v2.1.4^28^, with the best-suited nucleotide substitution model identified with ModelFinder. Support values were obtained with ultrafast bootstrapping with 1,000 replicates. The trees were visualized and annotated via Interactive Tree of Life (iTOL v6)^29^.

## Results

### Environmental sources of isolates and species distribution

**Fig. 1:**
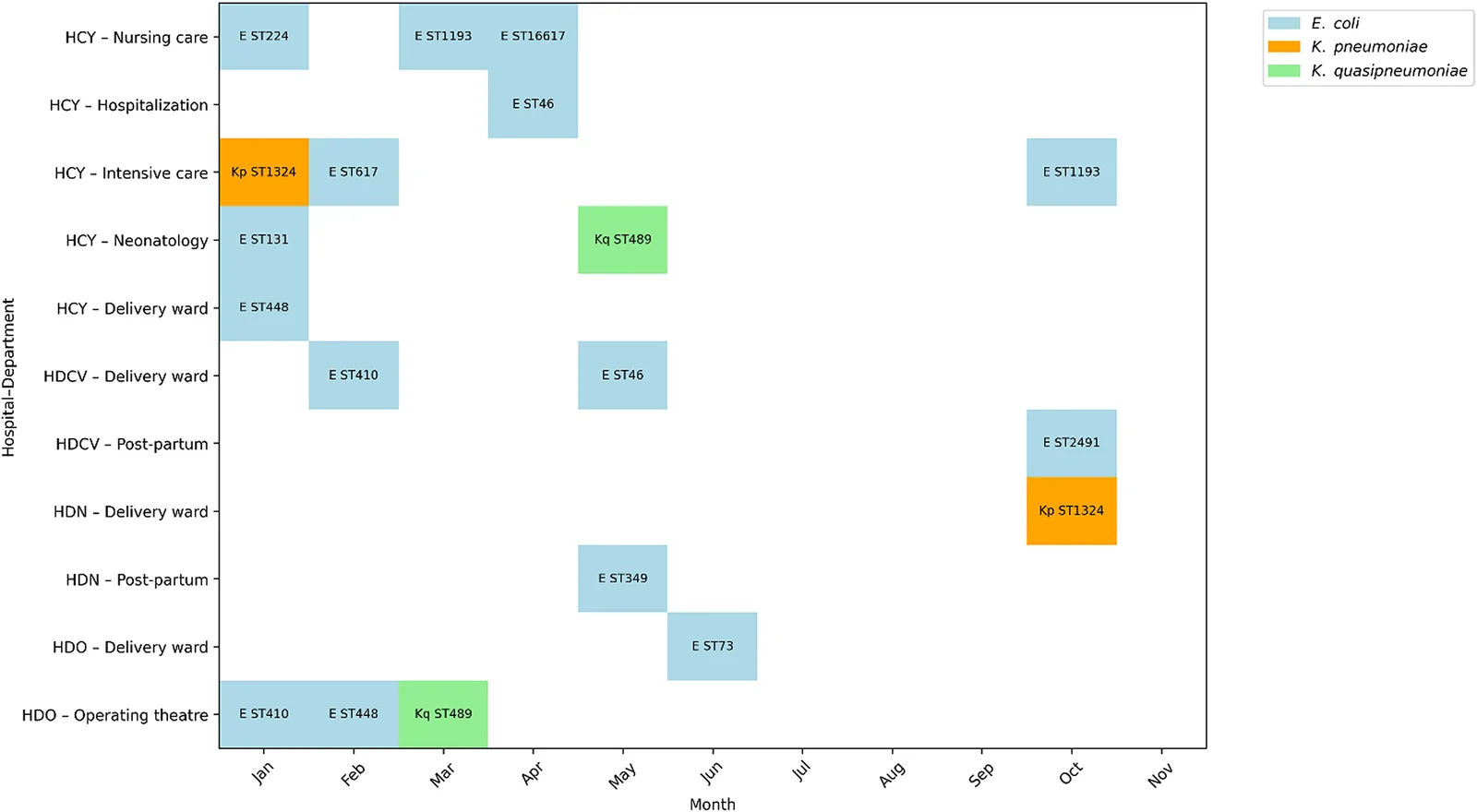
Heatmap showing monthly distribution of ESBL-producing *E. coli* and *Klebsiella* spp. across hospitals and maternity departments. E: *E. coli*; Kp: *K. pneumoniae*; Kq: *K. quasipneumoniae. HCY*: Yaounde Central Hospital; *HCV*: Cite verte district hospital; *HDN*: Nkolndongo district hospital; *HDO*: Odza district hospital.

From the 1519 environmental samples collected during the study, including 231 air samples and 1,288 surface swabs, 19 ESBL-producing *E. coli* and *Klebsiella* spp. isolates were obtained from surfaces across the four investigated maternity wards. The air-exposed plates did not yield any ESBL-producing *E. coli* or *Klebsiella* spp. The 19 strains consisted of fifteen isolates of *E. coli* and four *Klebsiella* spp., including two *K. pneumoniae* and two *K. quasipneumoniae* isolates. The highest number of isolates (n = 12; ten *E. coli* and two *K. pneumoniae*) were found at HCY, followed by HDO (n = 4; three *E. coli* and one *K.a pneumoniae*), HDCV (n = 3; three *E. coli*), and HDN (n = 2; one *E. coli* and one *K. pneumoniae*) (Fig. 1).

From a spatial perspective, isolates predominantly came from high-contact maternity environments such as intensive care, neonatology, delivery wards, hospitalization units, and operating theatres, whereas observation areas, offices, and several postpartum and newborn care units did not yield any isolates. From a temporal perspective, ESBL-producing isolates were detected sporadically during the study period, with the continuous presence of clinically significant *E. coli* STs (ST131, ST410, ST617, ST1193, ST73, and ST448), *K. pneumoniae* ST1324 and *K. quasipneumoniae* ST489 in several departments. In summary, ESBL-producing *E. coli* and *Klebsiella* spp. were mainly found on surfaces and were more commonly detected at tertiary facility (HCY) than at district hospitals.

### Antimicrobial susceptibility profiles

**Fig. 2:**
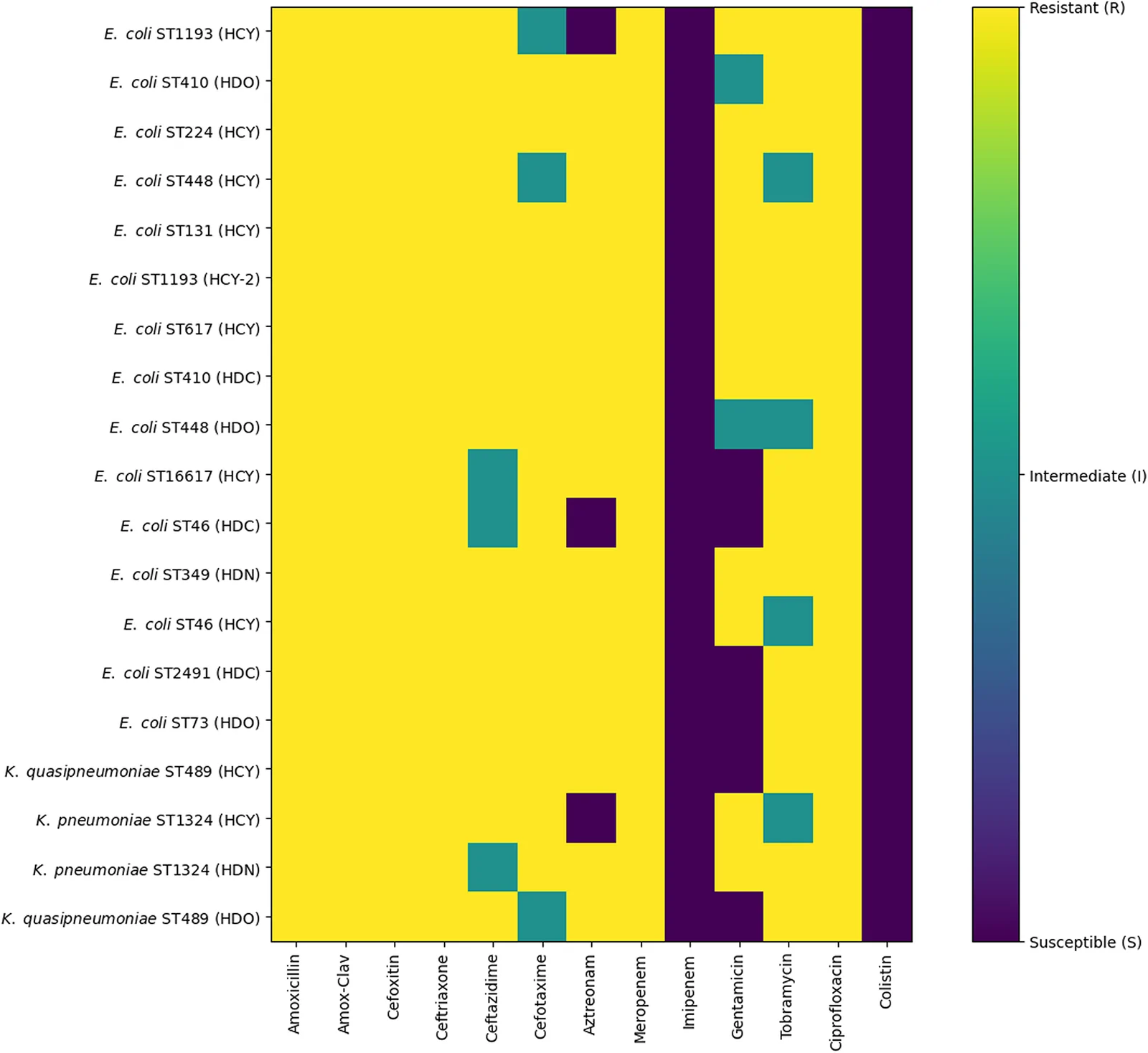
Heatmap of antimicrobial susceptibility profiles of ESBL-producing *Escherichia coli* and *Klebsiella* spp. isolates recovered from environmental surfaces of maternity wards in Yaoundé, Cameroon. Susceptibility results are shown for β-lactams, aminoglycosides, fluoroquinolones, and polymyxins. Colors represent resistant (R), intermediate (I), and susceptible (S) phenotypes. Absolute S/R/I classifications underlying this heatmap are provided in Supplementary Table S1.

All nineteen isolates had a multidrug-resistant (MDR) phenotype (Table S1) and were resistant to at least eight of the thirteen antibiotics used, which covered four drug classes. Overall, 18 out of 19 strains were resistant to ten or more antibiotics. All the strains were resistant to amoxicillin, amoxicillin-clavulanic acid and ciprofloxacin. There was universal resistance to third-generation cephalosporins, including ceftriaxone, ceftazidime, and cefotaxime, as well as cefoxitin. Resistance to aztreonam occurred in sixteen isolates, while three isolates were susceptible. Testing for carbapenem susceptibility revealed a disparity between the two antibiotics evaluated. All 19 isolates were resistant to meropenem but susceptible to imipenem. Many isolates were resistant to aminoglycosides: tobramycin resistance was observed in fifteen isolates, whereas gentamicin resistance was found in eleven isolates. Six isolates were sensitive to gentamicin, whereas two isolates were susceptible to increased exposure. Sensitivity to colistin was maintained in all isolates. The susceptibility profiles of the isolates are summarized in a heatmap (Fig. 2).

### Sequence types of *Escherichia coli* and *Klebsiella* species

MLST analysis revealed that *E. coli* and *Klebsiella* isolates from maternity ward surfaces presented high genetic diversity, and more than one *E. coli* ST, as well as *Klebsiella* ST, coexisted in maternity hospitals at different times (Fig. 1). *E. coli* isolates belonged to various sequence types (STs), such as the globally spread ST1193, ST410, ST131, and ST617 and the occasionally isolated STs ST224, ST448, ST349, ST46, ST73, ST2491, and ST16617. Some of the STs, including ST410 (2), ST46 (2), and ST448 (2), were found in more than one hospital. ST1193 was isolated twice in HCY, in March and August, whereas some were isolated in only one hospital. The *Klebsiella* isolates were classified into two species and sequence types: the two *K. pneumoniae* isolates belonging to ST1324 were isolated from two hospitals, as were the two *K. quasipneumoniae* isolates belonging to ST489.

### Phylogenetic relationships of *E. coli* isolates

**Fig. 3:**
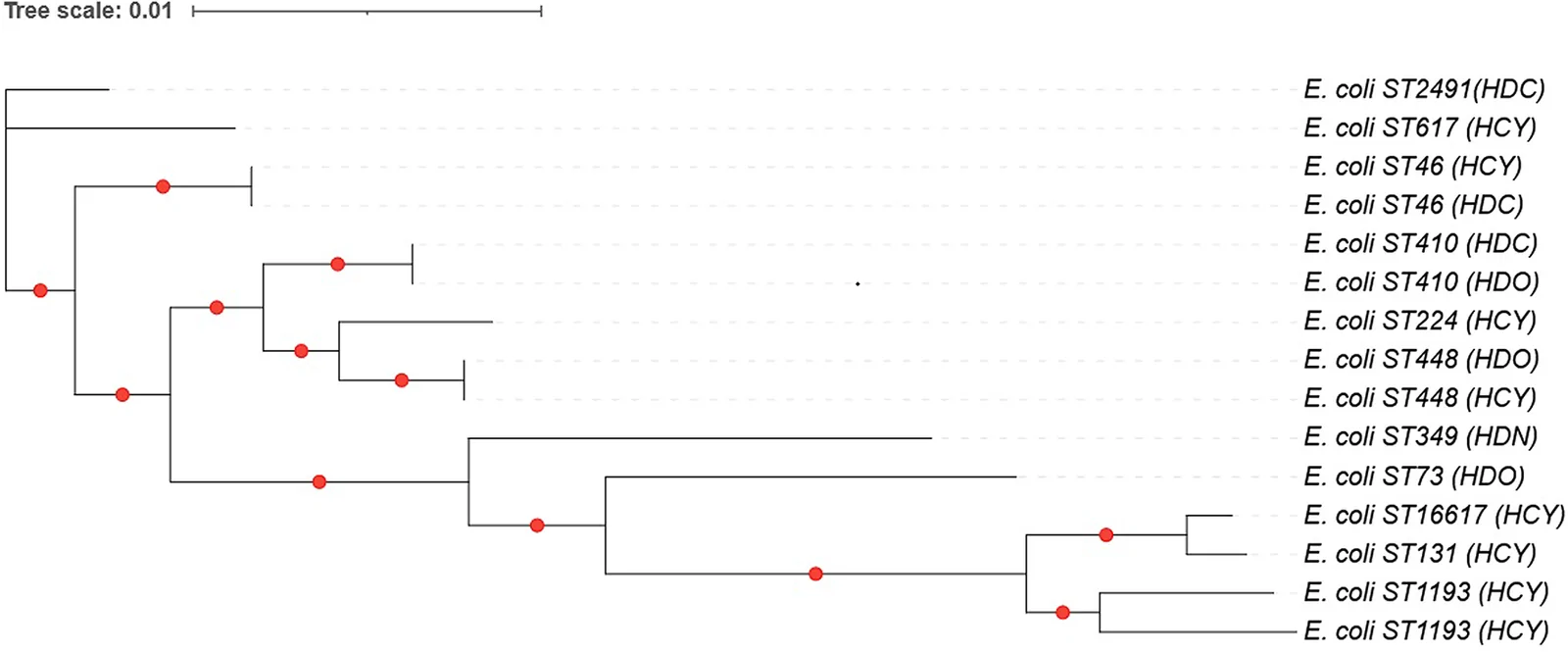
Core-genome maximum-likelihood phylogenetic tree of ESBL/carbapenemase producing *Escherichia coli* isolates recovered from maternity ward environments in Yaoundé, Cameroon. The tree illustrates the genetic relatedness of environmental *E. coli* isolates and the distribution of high-risk lineages circulating across different maternity wards. Bootstrap values ≥ 70% are marked with red dots at key nodes.

Core genome phylogenies were computed for the fifteen *E. coli* isolates, which were clustered according to sequence type (Fig. 3).

The cluster of ST1193-related isolates was tight and contained two isolates from the same maternity ward (HCY). The ST73 isolate grouped closely with these ST1193 isolates, while the ST131 isolate formed a separate lineage but was still within the same major clade. A separate isolate of ST16617 fell in a separate lineage next to ST131. ST410 isolates from two maternity wards clustered together with other isolates, forming a large clade. The ST46 isolates came from two wards and formed a cluster. The two ST448 isolates from different wards were so closely related that they formed a subcluster. In contrast, isolates of the ST224, ST349, ST617 and ST2491 STs were located on distinct branches of the phylogenetic tree.

### Plasmid-antibiotic resistance gene associations

**Fig. 4:**
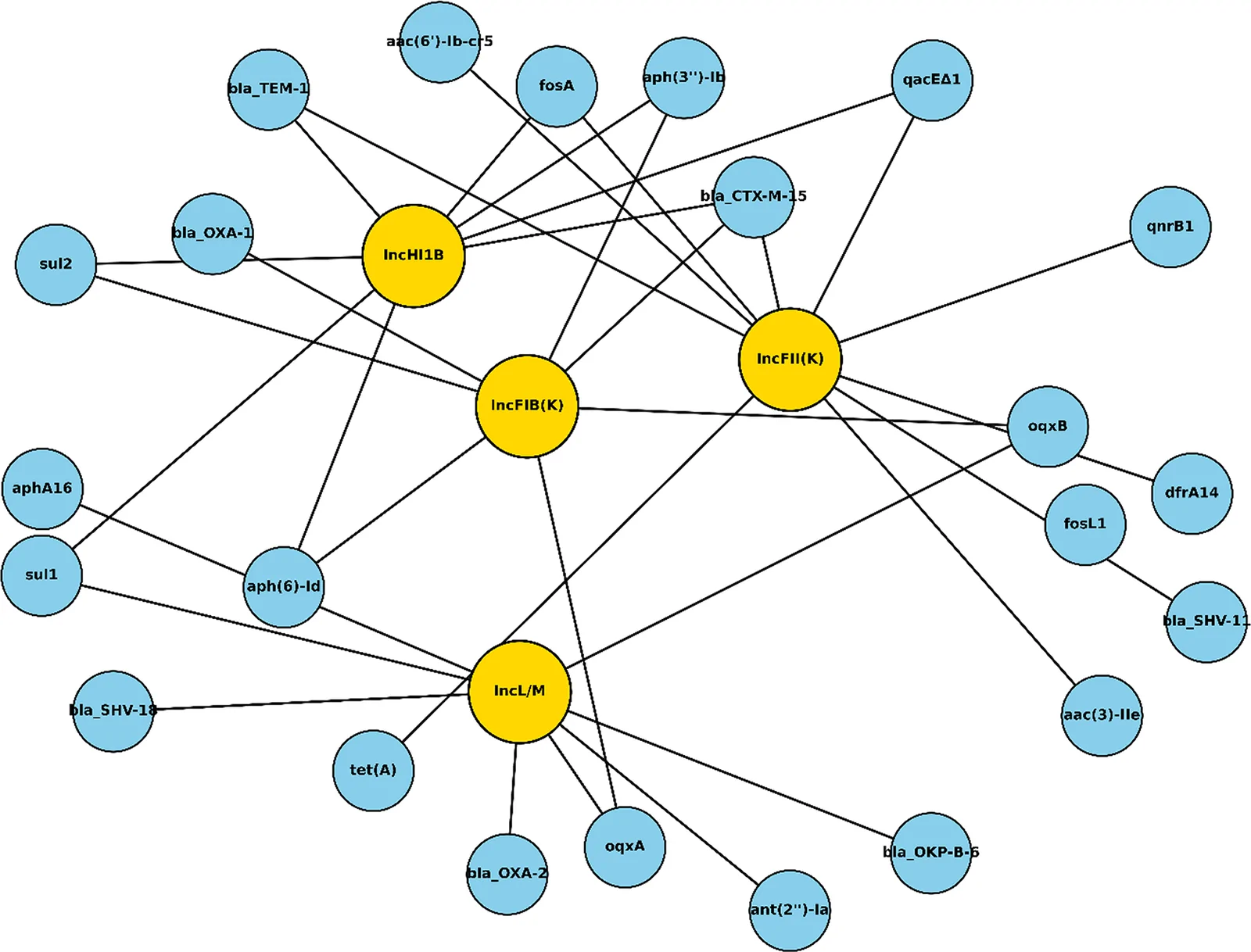
Plasmid-antibiotic resistance gene (ARG) association network in ESBL/carbapenemase producing *Klebsiella* spp. isolates recovered from maternity ward environmental surfaces in Yaoundé, Cameroon. Nodes represent plasmids (yellow) or resistance genes (blue), and edges indicate co-occurrence within the same isolate. The network highlights the central role of IncFII(K), IncFIB(K), IncL/M, and IncHI1B (pNDM-MAR) plasmids in carrying multiple β-lactam, aminoglycoside, fluoroquinolone, sulfonamide, fosfomycin, and disinfectant resistance determinants.

WGS revealed that the ESBL-producing *E. coli* and *Klebsiella* spp. isolates harboured highly diverse plasmid replicons and ARGs. Multiple replicons (plasmid incompatibility groups) associated with ARGs were identified. Replicons found in *Klebsiella* species included IncFII(K), IncFIB(K), IncL/M, and IncHI1B (pNDM-MAR), and each isolate had between two and three replicons. These isolates were associated with various plasmid-associated ARGs, including ESBL genes (*bla*_CTX-M-15_, *bla*_SHV-11_, *bla*_SHV-18_, *bla*_OKP-B-6_), carbapenemase-associated genes (*bla*_OXA-2_), and additional resistance determinants targeting aminoglycosides, fluoroquinolones, sulfonamides, fosfomycin, chloramphenicol, and disinfectants (Fig. 4).

**Fig. 5:**
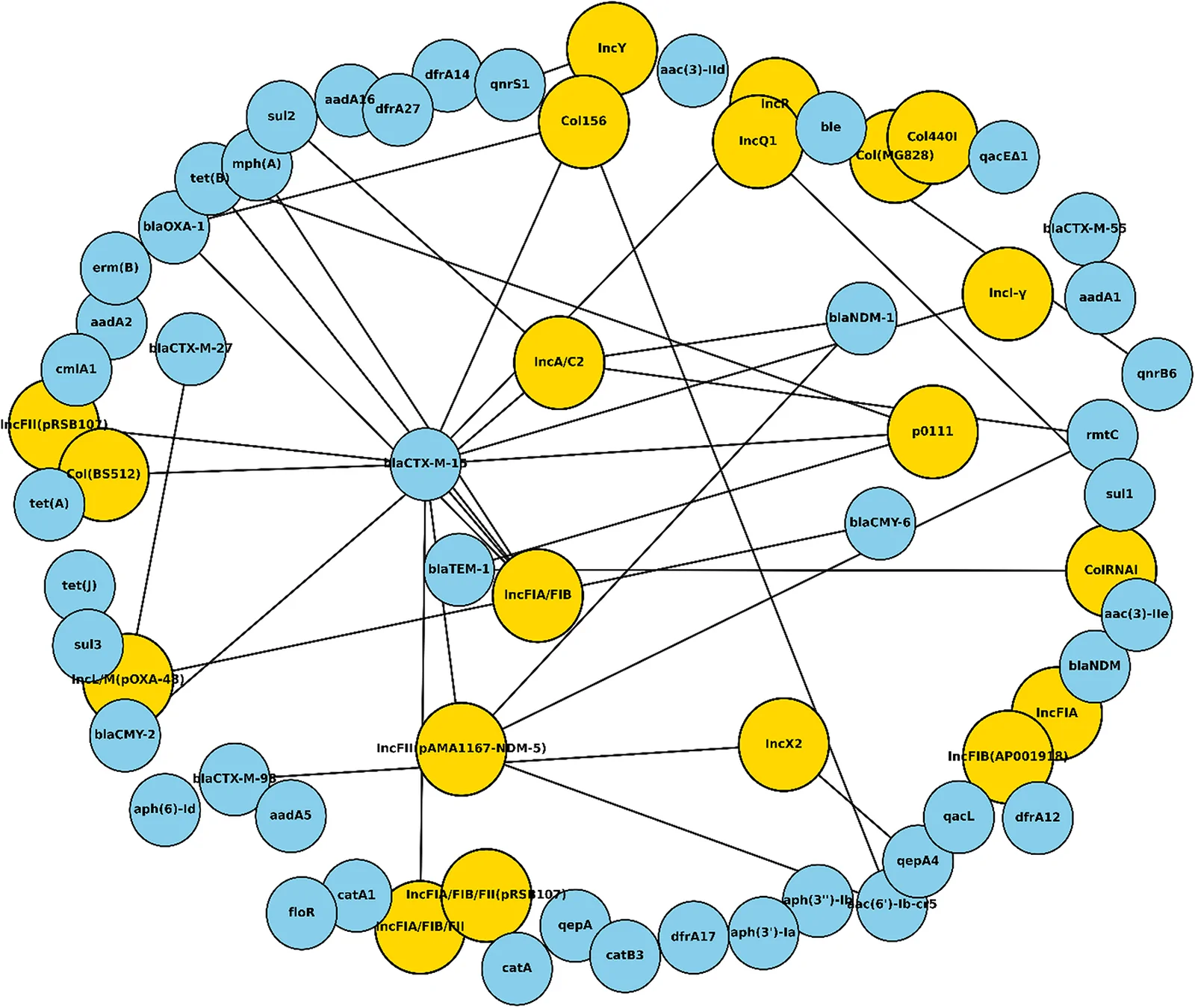
Plasmid-antibiotic resistance gene (ARG) association network in ESBL/carbapenemase producing *E. coli* isolates recovered from maternity ward environmental surfaces in Yaoundé, Cameroon. Nodes represent plasmids (yellow) or resistance genes (blue), and edges represent co-occurrence within the same isolate. The network highlights a dominant IncF plasmid backbone associated with ESBL genes, alongside broad-host-range plasmids (IncA/C2 and IncL/M) carrying carbapenemase and multidrug resistance determinants.

*E. coli* isolates carried a wide range of plasmid replicons, including IncFIA, IncFIB, IncFII, IncA/C2, IncL/M, IncX2, IncR, IncQ1, IncY, and several Col plasmids. Each isolate contained between one and 5 replicons. Epidemic plasmids such as IncFII (pAMA1167-NDM-5) and IncL/M (pOXA-48) were identified in various STs. Strong associations were found between IncF family plasmids (IncFIA, IncFIB, IncFII), IncA/C2 and IncL/M with ESBLs (*bla*_CTX-M-15_, *bla*_CTX-M-27_, *bla*_CTX-M-55_, *bla*_CTX-M-98_); AmpC β-lactamases (*bla*_CMY-2_, *bla*_CMY-6_); carbapenemases (*bla*_NDM-1_, *bla*_OXA-48_); aminoglycoside; macrolide, tetracycline, sulfonamide, trimethoprim, and chloramphenicol resistance genes; plasmid-mediated quinolone resistance determinants; and disinfectants, whereas IncX2, IncQ1, IncY, and Col plasmids presented more limited associations with specific subsets of resistance determinants (Fig. 5).

### Virulence factor profiling

Diverse determinants of virulence were found in both species. The four ESBL-producing *Klebsiella* spp. isolates presented a highly conserved set of virulence genes (Table 1).

**Table 1:** Functional classification of virulence determinants identified in ESBL-producing *Klebsiella* spp. isolates recovered from maternity ward environmental surfaces in Yaounde hospitals.

Functional category	Virulence determinants	*K. pneumoniae* ST1324 (HDN)	*K. pneumoniae* ST1324 (HCY)	*K. quasipneumoniae* ST489 (HDO)	*K. quasipneumoniae* ST489 (HCY)
Adhesins / Biofilm formation (ECP operon)	*ykgK/ecpR, yagZ/ecpA, yagY/ecpB, yagX/ecpC, yagW/ecpD, yagV/ecpE*	✓	✓	✓	✓
Iron acquisition (Enterobactin system)	*entA, entB, fepC*	✓	✓	✓	✓
Outer membrane proteins	*ompA*	✓	–	✓	✓
Motility / flagellar assembly	*fliQ*	–	✓	–	–

Presence of virulence determinants was inferred from whole-genome sequencing data. ✓ indicates presence of at least one gene within the functional category; – indicates absence.

The *ecp* operon genes, genes involved in iron acquisition mediated by enterobactin (*entA*, *entB*, and *fepC*), adhesion or biofilm formation, were present in all the isolates. The outer membrane protein *OmpA* was present in three isolates; the flagella-related gene *fliQ* was detected in only one isolate (*K. pneumoniae* ST1324 isolated at HCY).

The 15 isolates of *E. coli* presented a complex but organized virulence profile characterized by functions linked to persistence and host interaction (Table 2).

**Table 2:** Functional classification of virulence determinants identified in the 15 ESBL-producing *E. coli* isolates recovered from maternity ward environmental surfaces in Yaoundé hospitals.

Functional category	Representative virulence determinants	ST349 (HDN)	ST410 (HDO)	ST224 (HCY)	ST46 (HDC)	ST617 (HCY)	ST16617 (HCY)	ST131 (HCY)	ST448 (HDO)	ST1193 (HCY-1)	ST1193 (HCY-2)	ST410 (HDC)	ST2491 (HDC)	ST46 (HCY)	ST73 (HDO)	ST448 (HCY)
Adhesins / fimbriae / curli	*fim, pap, sfa/foc, ecp, csg, upaH*	✓	✓	✓	✓	✓	✓	✓	✓	✓	✓	✓	✓	✓	✓	✓
Iron acquisition / siderophores	*ent, fep, chu, yersiniabactin, iuc/iut*	✓	✓	✓	✓	✓	✓	✓	✓	✓	✓	✓	✓	✓	✓	✓
Capsule / serum resistance	*kps, iss*	✓	–	–	✓	–	–	✓	✓	✓	✓	–	–	–	✓	✓
Secretion systems	T2SS, T3SS, T6SS (*esp, eiv, clpV*)	✓	✓	✓	–	✓	✓	✓	✓	✓	✓	✓	✓	–	✓	✓
Toxins	*hlyE, cnf1, astA,* colibactin	✓	–	–	–	–	–	–	–	–	–	–	–	✓	✓	–
Motility / flagella	flagellar genes	✓	✓	✓	✓	–	✓	✓	✓	✓	✓	✓	✓	✓	✓	✓
Invasion / endothelial crossing	*ibeB/C*	✓	✓	✓	✓	✓	✓	✓	✓	✓	✓	✓	✓	✓	–	✓
Autotransporters / biofilm	*sat, vat,* biofilm-associated genes	–	✓	✓	–	✓	–	–	–	✓	✓	–	–	–	–	✓

Presence of virulence determinants was inferred from whole-genome sequencing data. ✓ indicates presence of at least one gene within the functional category; – indicates absence.

Adhesin genes, biofilm secretion system-related genes, iron acquisition system genes, and motility genes were represented in the majority of the isolates. The *ebeB/C* genes involved in invasion and secretion systems were widely distributed. The highly virulent extraintestinal pathogenic *Escherichia coli* (ExPEC) clades associated with ST131, ST73, ST1193, and ST410 presented an organized virulence profile that included adhesins, iron uptake systems, and virulence genes linked to capsule/serum resistance. The genes linked to toxins and autotransporters were limited to a subset of isolates.

### Co-occurrence of antimicrobial resistance and virulence determinants

Cross-referencing ARG profiles with virulence factor data for all 19 isolates revealed the co-occurrence of resistance and virulence determinants, as summarized in Supplementary Table S2. The *E. coli* isolates belonging to ST131, ST1193, ST410, and ST73 carried both multiple antimicrobial resistance genes and a broad range of virulence-associated genes. These included ESBL genes (*bla*_CTX-M-15_*, bla*_CTX-M-27_), carbapenemase genes (*bla*_NDM-1_*, bla*_OXA-48_), and plasmid-mediated quinolone resistance determinants, together with genes involved in iron acquisition, adhesion, invasion (*ibeB/C*), capsule/serum resistance (*kps, iss*), and secretion systems (T2SS, T3SS, T6SS). The ST73 isolate carried ESBL genes, multiple additional resistance determinants, and a virulence profile that included the colibactin gene cluster. Iron acquisition genes were detected in all 19 isolates of both species and co-occurred with antimicrobial resistance genes. Virulence gene profiles varied across sequence types. Isolates belonging to ST131, ST1193, ST73, and ST448 carried a broader range of virulence-associated genes compared to isolates assigned to ST224, ST349, ST2491, and ST16617. Plasmid replicon analysis showed that IncF family plasmids were present in multiple *E. coli* isolates and were associated with the presence of both ARGs and virulence-associated genes (Figs. 4 and 5).

## Discussion

This study shows that maternity ward environments in Yaounde, Cameroon, are consistently colonized by a diverse collection of MDR Enterobacterales, including globally distributed high-risk lineages of *E. coli* and *Klebsiella* spp. All isolates analyzed in this study were recovered exclusively from environmental surfaces, as no clinical isolates were included in the study design, with none detected in air samples, indicating predominantly surface-associated contamination. Contamination was highest in the tertiary hospital (HCY), with lower recovery in district hospitals (HDO, HDC, HDN), suggesting a gradient across healthcare levels.

By combining phenotypic antimicrobial susceptibility testing with WGS, we characterized ARGs, plasmid-mediated dissemination, virulence potential, and genetic relatedness of ESBL-producing isolates. These pathogens were recovered from high-contact surfaces in intensive care, neonatal and delivery wards, and operating theatres. This highlights the hospital environment as an important reservoir of antibiotic resistance and a source of exposure for vulnerable patients. Previous studies have shown that sinks, bed rails, work surfaces, and medical equipment can sustain long-term contamination with drug-resistant Gram-negative bacteria, facilitating indirect transmission^14–17^. The repeated recovery of ESBL-producing isolates across departments suggests sustained contamination. This likely reflects both environmental persistence and ongoing reintroduction. Such contamination is particularly concerning in maternity units, where neonates and postpartum mothers are highly vulnerable and contact intensity is high^18^.

The detection of globally distributed sequence types, including *K. pneumoniae* ST1324 *and K. quasipneumoniae* ST489 and *E. coli* ST131, ST1193, ST410, ST448, ST617, and ST73, suggests multiple introductions and ongoing circulation rather than a single outbreak. These clones are well known for their ability to acquire and disseminate ARGs through epidemic plasmids and are frequently associated with healthcare-associated infections^19–21^.

As expected, core-genome phylogeny was primarily structured by sequence type. STs represent stable evolutionary lineages rather than short-term transmission clusters^30^. Because ward metadata were not included in tree construction, clustering reflects genomic similarity rather than spatial proximity. In *E. coli*, no universal SNP threshold defines transmission. Lower SNP distances suggest recent spread, whereas higher distances (e.g., ≥ 100 SNPs) indicate unrelated isolates^31,32^. Therefore, genomic similarity must be interpreted alongside epidemiological context. The presence of related strains across wards may reflect transmission via staff, shared equipment, or patient movement. Alternatively, wards may represent multiple points of contact with larger hospital or community reservoirs^31,32^. These scenarios cannot be distinguished in a cross-sectional study without temporal data.

These findings reinforce the need for strengthened IPC measures, including hand hygiene and disinfection of high-touch surfaces^33,34^. Genomic surveillance could be integrated into AMR monitoring through periodic sequencing of MDR isolates. This would complement phenotypic testing and enable early detection of high-risk clones, in line with World Health Organization (WHO) and Africa Centres for Disease Control and Prevention (Africa CDC) recommendations^35^.

Comparison of phenotypic and genomic resistance showed high concordance (> 90%) for β-lactams, fluoroquinolones, and aminoglycosides. No phenotypic resistance lacked supporting genetic determinants. Most discrepancies involved carbapenems. Resistance to penicillins and third-generation cephalosporins is explained by ESBL genes, mainly *bla*_CTX-M-15_, which remains globally dominant and is strongly associated with successful ExPEC lineages^36,37^. Cefoxitin resistance in all isolates was linked to plasmid-mediated AmpC (*bla*_CMY_)^38^. Fluoroquinolone resistance was explained by plasmid-mediated genes and lineage-associated chromosomal mutations, particularly in ST131 and ST1193^39,40^. Aminoglycoside resistance corresponded to aminoglycoside-modifying enzyme genes.

Carbapenem discrepancies likely reflect differences in enzyme activity and bacterial genetic background. The pattern of imipenem resistance with preserved meropenem susceptibility is consistent with the carbapenemase genes identified in this study. Structural and kinetic analyses have shown that OXA-48 hydrolyzes imipenem more efficiently than meropenem. The impaired breakdown of meropenem, compared to imipenem, is most likely linked to the steric effect of meropenem’s 1β-methyl group on the deacylation reaction^41^. This specific methyl group provides a degree of protection against the Class D serine β-lactamases by hindering the rate-limiting deacylation step within the enzyme’s active site^42^. In addition, variations in NDM-1 expression and alterations in outer membrane permeability may further account for the differential susceptibility profiles observed^43^. The synergy between reduced porin expression, which limits antibiotic entry, and the specific hydrolytic efficiency of the enzymes present creates a complex resistance phenotype that can vary significantly between different carbapenem molecules^44^.

The detection of carbapenemase-producing Enterobacterales on maternity ward surfaces in Yaoundé despite the limited routine use of carbapenems in Cameroon can be explained by three non-mutually exclusive mechanisms. First, co-selection is likely a major driver. Carbapenemase genes such as *bla*_NDM-1_ and *bla*_OXA-48_ frequently reside on epidemic plasmids (IncF, IncL/M, and IncA/C2) that also carry resistance determinants against antibiotics in common use in Cameroon, including third-generation cephalosporins (*bla*_CTX-M-15_), fluoroquinolones (*qnr* genes), and aminoglycosides (*aac*, *aad*, *aph* genes)^45^. The heavy use of these other antibiotic classes may therefore indirectly sustain carbapenemase-encoding plasmids even in the absence of direct carbapenem pressure. Second, the global spread of high-risk clones may have introduced carbapenem resistance into local healthcare settings. The sequence types identified in this study, *E. coli* ST131, ST1193, ST410, and ST73, *K. pneumoniae* ST1324 and *K. quasipneumoniae* ST489, are internationally disseminated lineages that are well established in both community and hospital settings and may have acquired carbapenemase genes elsewhere before being introduced into hospitals in Yaoundé through travel, patient transfers, or community livestock interfaces^46^. Third, environmental persistence may allow these organisms to establish themselves long-term on hospital surfaces through biofilm formation and horizontal gene transfer, independently of ongoing carbapenem selection pressure^47^.

Therefore, these findings suggest that carbapenem resistance in this setting is likely sustained by co-selection through commonly used antibiotic classes and the importation of internationally disseminated high-risk clones, rather than by local carbapenem overuse alone^48,49^. The presence of these genes on maternity ward surfaces highlights silent environmental dissemination in high-risk settings.

The predominance of *bla*_OXA-48_ among the carbapenemase genes detected in our study is consistent with reported continental trends, in which *bla*_OXA-48_ accounted for approximately 42% of carbapenemase genes identified across African One Health studies^15^. The detection of IncF, IncL/M, and IncA/C2 plasmids supports their central role in regional spread^50^. Environmental data remain scarce, particularly in Cameroon and maternity settings. This study therefore fills an important gap and provides the first WGS-based evidence from such environments in Cameroon. The identification of high-risk clones further supports clone-driven dissemination across sub-Saharan Africa.

Similar trends have been reported in Nigeria^51^, Gabon^52^, Chad^53^, Ghana^54^, and Senegal^55^, where carbapenemase-producing Enterobacterales are increasingly found in both clinical and environmental settings. Together, these findings suggest that carbapenem resistance is becoming endemic in the region, driven by epidemic plasmids, cross-border transmission, and environmental reservoirs^49,50^. This underscores the need for coordinated regional surveillance.

These findings should be interpreted within a One Health framework. Human, animal, and environmental resistomes are interconnected, particularly in urban settings like Yaounde. Interactions between humans, livestock, and contaminated environments facilitate resistance transmission^56–58^. Resistant bacteria may enter healthcare settings via wastewater, food chains, or patients, and may return to the environment through inadequately treated waste^59,60^. This bidirectional exchange creates a self-reinforcing cycle and highlights the need for integrated AMR surveillance.

Therapeutic success depends primarily on phenotypic susceptibility, as clinical efficacy is determined by MIC values rather than the presence of resistance genes^61,62^. However, genomic detection remains essential for identifying carbapenemase producers with borderline phenotypes^38,63^. This study supports the combined use of phenotypic and genomic approaches. The accumulation of aminoglycoside-modifying enzyme genes (*aac, aad, aph*), often co-located with β-lactam resistance genes on MDR plasmids, likely explains the high prevalence of aminoglycoside resistance^64^. In contrast to the universal colistin resistance reported among clinical *K. pneumoniae* from hospitalized patients in Lagos, Nigeria^65^, all environmental isolates in our study remained susceptible to colistin. This difference may reflect the distinct source of isolation (environmental versus clinical), geographic variation in antimicrobial selective pressure, and the absence of *mcr* genes in our isolates, as confirmed by WGS^66^. However, its use in neonatal care remains limited due to toxicity and safety concerns.

Multiple plasmid replicons were identified, including IncF, IncA/C2, IncL/M, and IncHI1B, often carrying multiple resistance determinants. These plasmids are key drivers of global ESBL and carbapenemase dissemination^67–69^. Their presence in environmental isolates confirms hospitals as reservoirs of resistance plasmids. The detection of efflux systems (e.g., *oqxAB*) and disinfectant resistance genes (*qacEΔ1*) is concerning, as these may enhance persistence and reduce susceptibility to biocides^70^.

The isolates carried diverse virulence factors, including toxins, adhesins, capsule-associated genes, invasion genes, and iron acquisition systems, particularly in ExPEC lineages. These traits enhance colonization, persistence, and pathogenicity, including neonatal infections^71,72^. The detection of a colibactin gene cluster in an ST73 isolate is especially concerning given its role in host tissue damage^73^. The convergence of resistance, virulence, and persistence suggests a high potential for infection in vulnerable hosts.

Co-occurrence analysis showed that resistance and virulence are closely linked and concentrated in high-risk lineages, demonstrating that these traits converge rather than act independently. This reflects the genomic architecture of ExPEC clones, in which IncF plasmids co-carry resistance and virulence genes, enabling co-selection under antibiotic pressure^71,72^. Iron acquisition and adhesion/biofilm pathways were conserved across all 19 isolates of both *E. coli* and *Klebsiella* spp, indicating a shared core virulence strategy. These traits likely support both environmental persistence and invasive potential^74,75^. Concerns about the risks for maternal and newborn exposure in healthcare settings are heightened by the convergence of resistance, virulence, and environmental persistence traits, which implies that the environmental isolates from this study have the ability to colonize and possibly infect susceptible hosts.

The identified sequence types, specifically *E. coli* ST131, ST1193, ST410, and ST73, and *K. pneumoniae* ST1324, are globally recognized high-risk clones frequently implicated in urinary tract infections, neonatal sepsis, and hospital outbreaks^72^. These lineages carry a specialized “genomic toolkit” of virulence and carbapenemase genes characteristic of invasive ExPEC, which facilitates their transition from environmental reservoirs to clinical disease^46^. In sub-Saharan Africa, the regional circulation of these multidrug-resistant clones is a primary driver of maternal and neonatal mortality^48^. To definitively establish transmission links between these contaminated hospital surfaces and patient infections, future research must prioritize integrated environmental and clinical surveillance utilizing high-resolution WGS phylogenetic analysis^76^.

## Conclusions

To our knowledge, this is the first investigation where environmental sampling, phenotypic resistance testing, and WGS approaches are combined to examine ESBL and carbapenemase producers among Enterobacterales in maternity ward surfaces in Cameroon, as well as in Central Africa. In this study, the importance of maternity ward environments in Yaounde as potential sources of multidrug-resistant Enterobacterales was demonstrated, including epidemic lineages of *E. coli* and *Klebsiella* spp. The presence of epidemic ESBL and carbapenemase plasmids in different STs suggests the significance of hospital surfaces in sustaining and spreading AMR not only by direct contact between patients.

Considering that contamination of common surfaces may lead to indirect infection of extremely susceptible individuals, this research has important clinical implications related to maternal and neonatal care. Therefore, infection prevention and control interventions, especially environmental cleaning strategies, need to be implemented. This study addresses the crucial need for scientific data by presenting the first WGS-based genomic analysis of the environmental resistome in the maternity ward in Cameroon, thereby contributing to the existing literature on the region of Central Africa. The association between resistance genes and virulence factors, along with the predominance of iron uptake mechanisms and their clustering in highly virulent sequence types (ST131, ST1193, ST73, and ST410), suggests that these strains possess both the ability to persist and the potential for infection.

These findings support the integration of genomic surveillance into AMR monitoring frameworks and provide an evidence base for context-specific IPC strategies aimed at reducing the spread of multidrug-resistant pathogens in maternity settings in sub-Saharan Africa.

### Study limitations

The current study has certain limitations that need to be considered when the findings are interpreted.

First, it was not possible to screen staff members in healthcare settings or include clinical samples from mothers or newborns; only environmental surfaces could be tested for the presence of antibiotic-resistant bacteria. As a result, establishing a direct link between an isolate’s environmental presence and any subsequent colonization or infection of patients or employees was impossible.

Second, the small number of isolates, particularly *Klebsiella* species, makes it difficult to assess the diversity of the isolates and differences between wards, as it is unlikely that all the ESBL-resistant isolates present in the sampled wards were isolated in this study.

Third, a limited panel of antibiotics was used for antimicrobial susceptibility testing, and the minimum inhibitory concentration for each antibiotic was not assessed. This might have restricted the investigation of resistance levels, particularly with respect to colistin and carbapenems.

Fourth, long-read sequencing was not performed, which would have improved the assembly and examination of plasmid structures and links to resistance genes. Nevertheless, short-read sequencing has made it possible to thoroughly search for virulence factors, plasmids, and resistance genes.

Finally, systematic data on patient flow, cleaning procedures, antimicrobial use, and infection prevention strategies in maternity departments are lacking. This lack of context information made it more difficult to identify some of the contributing factors to environmental outbreaks.

Notwithstanding these drawbacks, the strength of combined phenotypic/genomic analysis is that it provides strong evidence of the diversity and presence of high-risk, multiresistant *Enterobacterales* in the maternity ward setting, prompting future investigations.

## Supplementary Information

Below is the link to the electronic supplementary material.

Supplementary Material 1.

## Data Availability

The datasets generated and/or analysed during the current study are available in the NCBI BioProject repository and support the findings of this study. They have been assigned the identifier [PRJNA1438821](https://www.ncbi.nlm.nih.gov/bioproject/1438821) and are publicly accessible at the following URL: (https://www.ncbi.nlm.nih.gov/bioproject/1438821).
